# Narrative review of non-pharmacological interventions for chemotherapy-induced peripheral neuropathy: exercise, physical therapy, cryotherapy and acupuncture

**DOI:** 10.3389/or.2026.1922088

**Published:** 2026-10-19

**Authors:** Lingjun Li, Mingsi Fan, Li Ni, Jing Wang, Li Qiao

**Affiliations:** 1 Oncology Department of Shanghai East Hospital Affiliated to Tongji University School of Medicine, Shanghai, China; 2 Medical Center of Stomatology, Shanghai East Hospital,School of Medicine, Tongji University, Shanghai, China; 3 Nursing Department, Shanghai East Hospital, Tongji University School of Medicine, Shanghai, China

**Keywords:** acupuncture, antineoplastic therapy, chemotherapy-induced peripheral neuropathy (CIPN), cryotherapy, exercise therapy, multidisciplinary care pathway, non-pharmacological interventions, peripheral neurotoxicity

## Abstract

**Purpose:**

Chemotherapy-induced peripheral neuropathy (CIPN) is a heterogeneous, treatment-limiting adverse event of cytotoxic chemotherapy that impairs sensory, motor, autonomic and balance function, and substantially reduces cancer patients’ quality of life. Current pharmacotherapy options are limited: duloxetine is the only guideline-recommended agent for established painful CIPN, while no drugs are approved for primary prevention. Non-pharmacological interventions have emerged as promising adjunctive strategies, yet evidence strength varies widely across modalities and patient subgroups. This narrative review aimed to synthesize contemporary clinical evidence of non-pharmacological interventions for CIPN prevention, symptom treatment and functional rehabilitation, to deliver stratified, chemotherapy-specific clinical guidance and identify gaps for future research.

**Method:**

A narrative review design was adopted, without pre-registration or standardized systematic review screening protocols. Targeted manual literature retrieval was conducted across PubMed, Web of Science, CNKI and Wanfang Data for peer-reviewed human clinical studies published between January 2018 and March 2025. We focused on four core non-pharmacological modalities for CIPN: multimodal exercise therapy, physical therapy incorporating cryotherapy, acupuncture-based complementary therapies, and behavioral self-management interventions. High-certainty evidence was defined as consistent results from large multicenter randomized controlled trials and network meta-analyses; moderate/low-certainty evidence was derived from small single-center trials and observational studies.

**Results:**

Cumulative stratified evidence demonstrates clear heterogeneity in intervention efficacy and clinical applicability. Multimodal supervised exercise and physical rehabilitation carry the most consistent high-certainty evidence for both primary CIPN prevention and functional recovery, with significant improvements in numbness, neuropathic pain, balance and muscle strength. Cryotherapy shows robust preventive efficacy exclusively in patients receiving taxane chemotherapy, with insufficient supportive data for platinum or vinca alkaloid regimens. Acupuncture, including warm needle acupuncture, TEAS and electroacupuncture, provides moderate-to-low certainty adjunctive relief for refractory established CIPN symptoms but is not recommended as first-line therapy. Behavioral interventions exert limited direct therapeutic effects on nerve injury, yet effectively improve symptom coping, treatment adherence and long-term self-management. All interventions exhibit favorable safety profiles with minimal severe adverse events when individualized to patient fitness, comorbidities and chemotherapy regimens.

**Conclusion:**

Non-pharmacological interventions cannot be considered uniformly effective for all CIPN populations; evidence strength and clinical indications differ markedly by modality. Supervised multimodal exercise and physical rehabilitation represent first-line adjunctive strategies for broad CIPN prevention and functional recovery. Cryotherapy is a targeted preventive option limited to taxane-treated patients. Acupuncture serves as secondary adjunctive care for persistent symptoms, while behavioral interventions function as supportive self-management tools. Clinical implementation requires agent-specific patient stratification, individualized intervention prescription, standardized validated CIPN assessment tools, and integrated multidisciplinary oncology rehabilitation pathways. Further large, stratified randomized controlled trials with long-term follow-up are required to standardize intervention protocols and validate sustained clinical benefits.

## 1 Introduction

As the incidence of malignant tumors continues to rise, cytotoxic chemotherapy remains an indispensable curative and palliative therapeutic strategy for multiple malignancies. Nevertheless, chemotherapy is accompanied by a broad spectrum of adverse toxicities, among which chemotherapy-induced peripheral neuropathy (CIPN) represents a highly prevalent, clinically debilitating complication. CIPN develops when cytotoxic agents inflict structural damage to peripheral sensory, motor and autonomic nerve fibers via peripheral neurotoxic mechanisms, producing a heterogeneous array of clinical manifestations such as bilateral acral numbness, muscular fatigue and bowel dysmotility that drastically erode patients’ health-related quality of life [1, 2]. Left unmanaged, severe CIPN may trigger sustained functional deficits and interrupt scheduled anti-cancer treatment delivery.

### 1.1 Clinical imperative for non-pharmacological CIPN interventions

Current pharmacological modalities for chemotherapy-induced peripheral neuropathy (CIPN) suffer from fundamental clinical deficits, which underscores the urgent clinical demand for standardized non-pharmacological supportive strategies. Per the authoritative American Society of Clinical Oncology (ASCO) clinical practice guidelines, duloxetine remains the sole pharmacotherapeutic agent endorsed for alleviating painful CIPN in patients with established neuropathy; no pharmaceutical agents have obtained guideline approval for primary CIPN prophylaxis [3]. This single first-line drug exhibits narrow therapeutic utility: it only targets neuropathic pain signaling pathways, with negligible ameliorative effects on core CIPN manifestations including distal paresthesia, limb motor weakness, gait instability and autonomic dysfunction, and it cannot reverse cumulative neurotoxic nerve damage or halt progressive functional deterioration over treatment cycles.

In addition to its limited symptomatic coverage, duloxetine carries a spectrum of systemic adverse reactions that compromise patient adherence among oncology cohorts. Common serotonergic-related toxicities include dizziness, refractory nausea, sleep disturbances and persistent asthenia, which compound chemotherapy-related physical distress and are poorly tolerated by frail patients with multiple chronic comorbidities. Beyond the suboptimal efficacy and unfavorable safety profile of duloxetine, untreated or undertreated CIPN imposes long-term irreversible functional risks on cancer survivors. Persistent neurotoxic injury induced by cytotoxic agents may become partially or permanently irreversible without timely intervention, elevating lifelong fall risks and impairing patients’ capacity to maintain independent activities of daily living for years following anti-tumor therapy completion. Clinically meaningful CIPN also acts as a major treatment-limiting barrier: moderate to severe peripheral neuropathy frequently forces clinicians to implement chemotherapy dose reductions, schedule delays or premature regimen discontinuation, which inevitably diminishes planned anti-tumor therapeutic intensity and impairs long-term oncologic prognosis.

Non-pharmacological rehabilitation interventions fill this unmet clinical gap by delivering dual preventive and rehabilitative benefits without systemic toxicities. Such multimodal regimens exert localized neuromuscular protective effects across sensory, motor and balance domains, which mitigate the severity of CIPN during chemotherapy administration and facilitate functional recovery once neurological symptoms emerge. By blunting the progression of chemotherapy-triggered peripheral nerve injury, tailored non-pharmacological protocols effectively lower the risk of treatment disruption, enabling patients to complete full-dose, scheduled cytotoxic cycles as originally planned and preserving the intended anti-neoplastic efficacy of curative or palliative chemotherapy regimens.

The clinical management of CIPN therefore constitutes a persistent clinical challenge owing to the paucity of viable pharmacological alternatives, which has driven expanding research focus on non-pharmacological therapeutic frameworks. Despite the limitations of available pharmacotherapy, an accumulating body of high-quality clinical evidence substantiates the favorable safety and consistent clinical benefits of structured non-pharmacological interventions—particularly multimodal exercise protocols—for both CIPN prevention and symptomatic relief [3]. This narrative review systematically synthesizes cutting-edge research advances regarding non-pharmacological interventions targeting CIPN, encompassing exercise therapy, physical therapy (including cryotherapy), acupuncture-based adjunctive modalities, behavioral interventions and integrated multidisciplinary care pathways. We differentiate the preventive, symptomatic and rehabilitative indications of each intervention category and stratify evidence certainty hierarchically, with the aim of generating standardized, comprehensive clinical references for non-pharmacological CIPN diagnosis and management.

### 1.2 Biological heterogeneity of chemotherapy-induced peripheral neuropathy by chemotherapeutic class

Chemotherapy-induced peripheral neuropathy exhibits profound biological and clinical heterogeneity, which is largely dictated by the specific class of neurotoxic cytotoxic agents administered, with distinct temporal patterns, symptomatic phenotypes, nerve injury reversibility, and mechanistic pathways observed across taxanes, platinum compounds, and vinca alkaloids. Taxane agents predominantly trigger early-onset, sensory-predominant CIPN characterized by cold allodynia and distal paresthesia; nerve damage arising from taxane exposure is largely reversible upon treatment discontinuation, with minimal persistent motor dysfunction reported. Platinum-based chemotherapies induce cumulative, dose-dependent large-fiber sensory loss that frequently persists long after chemotherapy completion, conferring a higher risk of permanent peripheral nerve deficits; their neurotoxic signature rarely manifests prominent motor impairment. By contrast, vinca alkaloids preferentially damage motor nerve fibers, resulting in early-onset muscle weakness, gait instability, and elevated fall risk, alongside milder sensory disturbances relative to platinum regimens. Such agent-specific heterogeneity necessitates stratified, chemotherapy-tailored non-pharmacological intervention plans, as preventive and rehabilitative modalities demonstrate inconsistent efficacy across these three major drug subgroups throughout existing clinical trials. This biological variability is emphasized repeatedly in subsequent intervention subsections and the manuscript’s Limitations chapter to underscore the importance of regimen-stratified clinical trial design and personalized supportive care delivery.

## 2 Methods

This manuscript adopts a narrative review methodology, without pre-registration, standardized PRISMA systematic review workflows, dual independent study screening, or comprehensive multi-database automated search strategies.

### 2.1 Literature search strategy

Targeted manual literature retrieval was performed across four electronic databases: PubMed, Web of Science, China National Knowledge Infrastructure (CNKI), and Wanfang Data. The search period covered 1 January 2018, to 31 March 2025. Combined English and Chinese search terms included: chemotherapy-induced peripheral neuropathy (CIPN), exercise, physical therapy, cryotherapy, acupuncture, transcutaneous electrical acupoint stimulation, behavioral intervention, rehabilitation, non-pharmacological management, supportive care.

### 2.2 Inclusion and exclusion

Criteria Inclusion criteria: (1) original human clinical studies, systematic reviews, meta-analyses, clinical expert consensus guidelines; (2) research evaluating non-pharmacological interventions for chemotherapy-induced peripheral neuropathy (CIPN) induced by cytotoxic chemotherapy; (3) full-text accessible peer-reviewed publications; (4) articles published in English or Chinese. Exclusion criteria: (1) animal experimental research, *in vitro* mechanistic studies without human clinical data; (2) single case reports, retrospective small case series; (3) neuropathy induced by targeted therapy, immunotherapy, proteasome inhibitors or immunomodulatory agents; (4) non-interventional observational descriptive studies without efficacy outcome data.

### 2.3 Evidence grading framework

Included literature was stratified into three evidence tiers based on study design, consistency of pooled results and overall risk of bias: (1) High-certainty evidence: Multicenter randomized controlled trials, network meta-analyses with consistent pooled outcomes and low heterogeneity; (2) Moderate-certainty evidence: Single-center randomized controlled trials, systematic reviews with moderate inter-trial heterogeneity; (3) Low-certainty evidence: Small non-randomized observational trials, retrospective cohort studies, expert consensus without quantitative clinical trial validation. All intervention efficacy conclusions in the manuscript are labeled with corresponding evidence tier for transparent clinical interpretation.

## 3 Non-pharmacological interventions for chemotherapy-induced peripheral neuropathy (CIPN)

### 3.1 Exercise therapy

Multimodal exercise regimens integrating aerobic, resistance, balance, and sensorimotor training deliver the most robust, consistent high-certainty evidence base for the non-pharmacological management of chemotherapy-induced peripheral neuropathy (CIPN) [49, 50][High-certainty]. Exercise interventions may enhance peripheral blood oxygenation, support partial damaged nerve repair, and relieve distal numbness and tingling; the exact molecular neuroprotective mechanisms remain incompletely characterized and require further translational validation [4]. A 2025 network meta-analysis by Wang et al. compared 19 distinct exercise protocols for CIPN and identified combined multimodal training as the optimal regimen to mitigate composite CIPN symptom burden, alleviate neuropathic pain, and improve balance and muscular strength [High-certainty]. Quantitative pooled analyses revealed distinct effect sizes across single and composite training modalities, as detailed in the following four independent subsections.

#### 3.1.1 Aerobic training

Primary clinical application: Primary prevention of mild CIPN and auxiliary functional rehabilitation for established mild-to-moderate neuropathy.

Regular aerobic physical training combined with sensorimotor practice significantly reduces global CIPN severity, with a standardized mean difference (SMD) of −1.06 (95% CI, −1.77 to −0.36)[High-certainty]. Isolated aerobic exercise demonstrates moderate efficacy for alleviating general sensory discomfort associated with CIPN; walking-based aerobic programs yield the largest pooled improvements in broad neuropathy symptoms (SMD = −2.07; 95% CI, −2.70 to −1.43) [High-certainty]. When paired with resistance and balance training, aerobic exercise generates amplified clinical benefits (SMD = −1.88; 95% CI, −2.81 to −0.94)[High-certainty]. For isolated CIPN-related neuropathic pain, hand-foot aerobic training produces prominent symptom relief (SMD = −1.99; 95% CI, −2.85 to −1.13)[High-certainty]. Aerobic training is well-tolerated across most cancer cohorts receiving vinca alkaloid or oxaliplatin chemotherapy, with minimal adverse physical burden [Moderate-certainty].

#### 3.1.2 Resistance training

Primary clinical application: Functional rehabilitation for CIPN-induced muscular weakness and pain relief.

Resistance training targets distal muscle atrophy and strength loss secondary to CIPN [High-certainty]. Combined aerobic-resistance exercise achieves a large pooled effect size for CIPN pain control (SMD = −1.13; 95% CI, −1.58 to −0.67)[High-certainty]. Strengthening-focused resistance regimens exert the most potent effects on CIPN-associated pain (SMD = −11.43; 95% CI, −13.60 to −9.26)[High-certainty]. Regular resistance practice preserves lean muscle mass and attenuates progressive motor impairment throughout multi-cycle chemotherapy, making it a core rehabilitative component for patients presenting established motor CIPN manifestations [Moderate-certainty].

#### 3.1.3 Balance training

Primary clinical application: Functional rehabilitation for CIPN-related gait instability and fall risk reduction.

Balance-focused training primarily addresses proprioceptive deficits and elevated fall risk driven by peripheral nerve damage [High-certainty]. When incorporated into combined exercise frameworks, balance work substantially improves postural stability and daily functional performance in CIPN patients. Meta-analytic data confirm that training protocols integrating balance components achieve consistent reductions in balance impairment severity, alongside concurrent relief of numbness and tingling symptoms [Moderate-certainty]. Balance training is recommended as a mandatory supplementary component for all high CIPN-risk groups, particularly elderly patients with frailty [Low-certainty].

#### 3.1.4 Multimodal sensorimotor training

Primary clinical application: Dual use for primary CIPN prevention and comprehensive functional rehabilitation of severe symptomatic neuropathy.

Supervised sensorimotor training (SMT) and whole-body vibration (WBV) constitute the most evidence-supported multimodal sensorimotor modalities [High-certainty]. A landmark multicenter randomized controlled trial by Streckmann et al. [5] demonstrated twice-weekly 15–30 min supervised SMT or WBV during chemotherapy drastically lowered incident CIPN risk relative to standard treatment-as-usual (TAU). Intention-to-treat (ITT) analysis recorded CIPN incidence of 30% (SMT group), 41% (WBV group), and 71% (TAU control group; P = 0.002), with the strongest preventive effect observed in patients receiving vinca alkaloids [High-certainty]. Per-protocol (PP) analysis requiring ≥75% intervention adherence yielded even more prominent risk reduction: CIPN incidence fell to 28.6% (SMT) and 37.5% (WBV). The intergroup incidence disparities are visualized in Figure 1.

**FIGURE 1:**
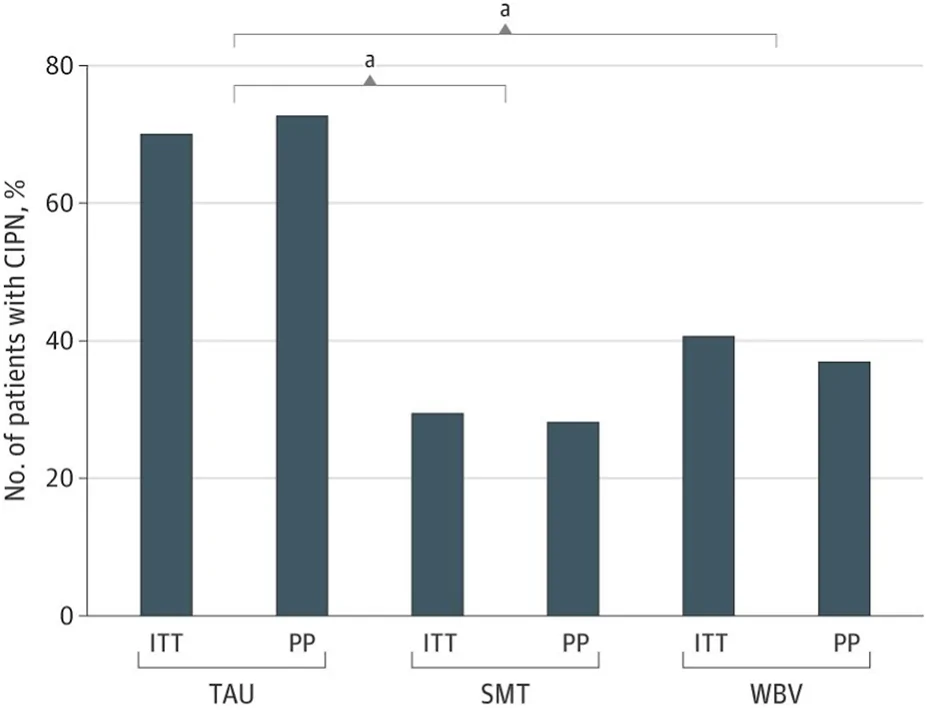
Proportion of patients with chemotherapy-induced peripheral neuropathy (CIPN) in the treatment-as-usual (TAU; usual care), spinal manipulative therapy (SMT), and whole-body vibration (WBV) groups under intention-to-treat (ITT) and per-protocol (PP) analyses. The marker a denotes a statistically significant between-group difference [5].

Multiple systematic reviews corroborate these outcomes. Crichton et al. [6] reported structured multimodal exercise significantly lowered CIPN severity (SMD −0.89; 95% CI, −1.37 to −0.41; p = 0.0003) and preserved physical function (SMD 0.51; 95% CI, 0.02 to 1.00; p = 0.04)[Moderate-certainty]. Subgroup analyses confirm consistent therapeutic gains across most tumor and chemotherapy subtypes, with maximal symptom mitigation among patients treated with vinca alkaloids or oxaliplatin [5, 7] [Moderate-certainty].

#### 3.1.5 Standardized exercise prescription

The optimal multimodal exercise prescription for CIPN prevention and rehabilitation integrates aerobic, resistance, balance, and sensorimotor training modules [High-certainty]. Evidence-based supervised protocols run 2–3 times weekly over 6–20 weeks, with single-session durations ranging from 15 to 45 min [5, 7, 8] [High-certainty]. The STOP trial reported 50%–70% relative CIPN incidence reduction via twice-weekly SMT/WBV delivered throughout chemotherapy [9][Moderate-certainty]. Higher weekly training frequency, longer intervention cycles and extended single-session duration correlate with greater improvements in CIPN severity, pain and balance dysfunction [High-certainty]. Another randomized trial demonstrated thrice-weekly combined sensorimotor and resistance training slowed sensory CIPN progression and sustained muscle strength in high-adherence participants [8]. Meta-analytic evidence confirms ≥2 weekly sessions, minimum 8-week intervention cycles, and extended weekly training time correlate with greater improvements in CIPN severity, pain, and balance dysfunction [7, 10]. Supervised in-clinic training generates superior clinical benefits compared to unsupervised home-based programs, and strict protocol adherence is required to attain maximal symptom relief [Moderate-certainty].

Exercise intensity must be individualized to each patient’s baseline fitness and comorbid burden. Low-intensity mind-body practices (Baduanjin, Wuqinxi, Tai Chi, Pilates) may be integrated as supplementary daily activity, paired with warm foot soaks and hand-foot Jiaji acupoint massage to boost peripheral circulation and ease CIPN numbness and pain [11] [Low-certainty]. Training load can be progressively elevated as patient tolerance improves [12]. Combined mind-body and sensorimotor training substantially lessens CIPN symptom severity and restores peripheral depth sensation [10]. A standardized multimodal exercise prescription framework is summarized in Table 1.

**TABLE 1:** Standardized multimodal exercise prescription scheme for CIPN prevention and management.

Component	Intensity	Duration	Frequency
Aerobic exercise (e.g., walking)	Moderate	20–30 min	2–3 times/week
Resistance training	Moderate	15–20 min	2–3 times/week
Balance exercises	Moderate	10–15 min	2–3 times/week
Sensorimotor training (e.g., proprioceptive tasks, WBV)	Moderate	15–30 min	2 times/week

WBV, Whole-Body Vibration.

#### 3.1.6 Safety, contraindications, and special populations

Exercise interventions are generally safe, with no serious adverse events reported in the literature [5–7] [High-certainty]. However, minor adverse events such as transient musculoskeletal discomfort or fatigue may occur but can be managed with appropriate supervision and individualized prescriptions [6, 7]. Absolute contraindications include acute medical instability, such as uncontrolled cardiac disease, acute infections, or severe thrombocytopenia with a bleeding risk. Relative contraindications include severe balance impairments, active foot ulcers, or musculoskeletal injuries that could worsen with exercise [5, 7]. In such instances, exercise protocols should be modified or postponed until the underlying condition is addressed. Special populations require tailored approaches; for example, older adults benefit from supervised, low-to-moderate intensity exercise, with particular attention paid to frailty, balance, and fall risk [5, 13]. Sensorimotor training and WBV are especially beneficial for this demographic [5] [Moderate-certainty]. Although pediatric data are scarce, exercise is presumed safe when adapted to the developmental stage and existing comorbidities. Furthermore, the current body of evidence supporting exercise interventions predominantly involves non-Chinese populations, which may differ significantly in cultural backgrounds, disease and treatment characteristics, and levels of physical fitness. Consequently, there is a compelling need for localized research [Low-certainty].

### 3.2 Physical therapy

Physical therapy refers to structured physical rehabilitation and external physical modality interventions for CIPN, covering customized movement training and localized cold physical intervention proposed a comprehensive clinical workflow for physical therapists, which includes detailed sensory and strength assessments, balance and gait analysis, and individualized sensorimotor, strength, balance, and endurance training, supplemented by home-based aerobic exercises [14]. Physical therapists are advised to adapt exercise plans based on patient progress, tolerance, and any adverse events encountered. Although no formal physical therapy guidelines specifically address CIPN, this framework is supported by neurorehabilitation literature and clinical trial data.

#### 3.2.1 Core physical rehabilitation framework

Standard physical rehabilitation relies on progressive sensorimotor and functional training to restore peripheral nerve sensitivity, muscle strength and balance function, which shares overlapping training logic with multimodal exercise therapy described in Section 2.1. Therapists prioritize real-time sensory testing to adjust training intensity for patients with numbness, tingling or movement limitation caused by CIPN.

#### 3.2.2 Standardized physical therapy clinical workflow

Clinicians conduct baseline sensory, motor and gait assessment before chemotherapy cycles, then formulate weekly supervised rehabilitation plans [Low-certainty]. Follow-up assessment is arranged after every two chemotherapy cycles to modify training items, reduce fall risk and slow nerve function deterioration. Home-based daily stretching and balance practice are prescribed as supplementary daily physical therapy to extend intervention effects between hospital visits [Moderate-certainty].

#### 3.2.3 Cryotherapy

Cryotherapy, which involves applying cold to the extremities during chemotherapy infusion, has emerged as an effective non-pharmacological strategy for preventing chemotherapy-induced peripheral neurotoxicity (mechanism-only usage), especially in breast cancer patients receiving taxanes, as it helps protect motor and sensory nerves [15] [High-certainty]. A 2025 network meta-analysis conducted by Li et al. [16] found that cryotherapy was significantly more effective at alleviating CIPN than usual care, with a standardized mean difference of −1.22 (95% CI, −2.26 to −0.17), outperforming both exercise and self-acupressure [High-certainty]. The Surface Under the Cumulative Ranking Curve (SUCRA) scores were as follows: cryotherapy 0.74, exercise 0.62, and self-acupressure 0.59. Additional meta-analyses and randomized trials [17, 18] support these findings, demonstrating a significant reduction in CIPN incidence and an improvement in quality of life [High-certainty]. For instance, Tai et al. [18] reported that cryotherapy reduced the incidence of CIPN with a risk ratio (RR) of 0.23 (95% CI, 0.13–0.43) at mid-chemotherapy and an RR of 0.54 (95% CI, 0.33–0.88) at the end of chemotherapy. Similarly, Hanai et al. [17] observed significantly lower rates of both objective and subjective CIPN symptoms in the treated extremity, with tactile sensitivity rates of 27.8% compared to 80.6% in the control group (odds ratio 20.00; P < 0.001).

Although generally considered safe, cryotherapy [3, 16, 18] still necessitates careful patient screening and close monitoring to prevent complications such as frostbite. Contraindications include pre-existing peripheral vascular disease, Raynaud’s phenomenon, cold urticaria, cryoglobulinemia, and the presence of open wounds, ulcers, or severe sensory loss in the extremities. The typical protocol involves the use of frozen gloves and socks or continuous flow cooling systems, starting 15 min before the chemotherapy infusion and continuing for 15 min afterward [18]. It is crucial that nursing staff supervise and educate patients about the signs of cold injury [Moderate-certainty]. Various preventive strategies have been described; one involves cooling the extremities with circulating pressure (5–15 mmHg) at a temperature of 16 °C for up to 3 h, while another employs a continuous flow cooling system with water at a temperature of 22 °C for a maximum of 4 h [19].

### 3.3 Complementary adjunctive therapies

This section covers two non-physical auxiliary interventions for persistent CIPN symptoms: acupuncture and behavioral self-management strategies. Unlike exercise and physical therapy (including cryotherapy) that serve as primary preventive and rehabilitative tools, the therapies below act as supplementary add-on treatments for patients with refractory sensory discomfort who fail to achieve full symptom relief via core physical interventions.

#### 3.3.1 Acupuncture therapy

The evidence supporting the efficacy of acupuncture in managing established CIPN appears promising. A multicenter randomized trial conducted by Ben-Arye et al. [20] demonstrated that acupuncture, whether accompanied by complementary integrative medicine or not, significantly ameliorated symptoms related to CIPN and enhanced physical function compared to standard care. This included improvements in hand numbness, tingling, pain, discomfort, and EORTC physical function scores (p < 0.05). Acupuncture reduces CIPN incidence in oxaliplatin and lung cancer chemotherapy cohorts [21, 22] [Moderate-certainty]. Wu et al. [21] randomized 60 patients undergoing oxaliplatin-based chemotherapy into three groups to evaluate the efficacy using the Levi sensory neurotoxicity score. The findings indicated a neurotoxicity incidence of 30.00% in the acupuncture treatment group, 36.84% in the Western medicine control group, and 78.95% in the chemotherapy-only group. Both the acupuncture and Western medicine groups outperformed the chemotherapy-only group. However, there was no statistically significant difference between the acupuncture and Western medicine groups (p > 0.0167). Neurotoxicity grading was lower in both the acupuncture and Western medicine groups compared to the chemotherapy-only group (p < 0.05), with no significant difference between the former two (p > 0.05). Manual acupuncture, warm-needle acupuncture and transcutaneous electrical acupoint stimulation (TEAS) generate mild to moderate relief of established CIPN sensory symptoms [Moderate-certainty]. In a randomized controlled trial, Pan et al. [22] involved 300 subjects and assessed the effect of transcutaneous electrical acupoint stimulation (TEAS) at five specific acupoints, Hegu, Quchi, Zusanli, Sanyinjiao, and Taichong, on reducing the overall incidence of CIPN in lung cancer patients. Warm-needle acupuncture combined with moxibustion improves CIPN severity and quality of life relative to neurotropin injection [23] [Low-certainty]. Tian et al. [23] allocated 76 cancer patients with chemotherapy-induced peripheral neurotoxicity into either a warm-needle acupuncture group or a control group, with 38 participants in each. The treatment group received warm-needle acupuncture combined with moxibustion at specific bilateral acupoints, with needles retained for 30 min. Warm-needle acupuncture combined with moxibustion improves CIPN severity and quality of life relative to neurotropin injection [23] [Low-certainty]. During this period, moxa sticks were affixed to the needles at Hegu, Waiguan, Yanglingquan, and Zusanli for moxibustion, utilizing three moxa cones per needle. The control group was administered intramuscular injections of Neurotropin at the same acupoints. After one treatment course, significant improvements in neurotoxicity grading and quality of life were observed in the warm-needle acupuncture group compared to the control group (p < 0.05). Systematic reviews [24] have shown that both acupuncture and exercise can mitigate neuropathic pain, although their impact on quality of life is still uncertain. According to ASCO guidelines [3], acupuncture is considered reasonably safe and may be proposed as an adjunctive treatment [Low-certainty]. However, due to the low certainty of evidence, it is not recommended as a primary therapy.

When performed by adequately trained practitioners, acupuncture is generally considered safe. Minor local reactions, such as bruising or soreness, may occur; however, serious complications are infrequent [20, 25]. Contraindications to acupuncture include severe neutropenia or thrombocytopenia, local infection or ulceration at the intended needling site, and needling in limbs affected by lymphedema [25]. Practitioners experienced in oncology should perform acupuncture. Furthermore, patients with diabetes or peripheral vascular disease require vigilant monitoring for foot ulcers and should avoid cryotherapy [6, 16].

#### 3.3.2 Behavioral interventions

Behavioral interventions, which include education, self-management resources, and symptom self-reporting, have demonstrated preliminary benefits in alleviating symptoms of CIPN and enhancing patient outcomes. However, the certainty of this evidence remains low [6, 26] [Low-certainty]. These interventions should be employed as supplementary treatments due to the uncertainty surrounding the magnitude and durability of their benefits. Effective translation of evidence into clinical practice necessitates precise quantitative recommendations concerning the dose, frequency, duration, safety profiles, contraindications, and patient selection of interventions, with particular focus on vulnerable populations.

## 4 Practical multidisciplinary team (MDT) collaborative care pathway

### 4.1 The successful implementation of non-pharmacological interventions for CIPN critically depends on their integration into multidisciplinary oncology care pathways

ASCO guidelines and expert frameworks underscore the importance of early identification of high-risk patients, comprehensive assessment, tailored intervention planning, and continuous monitoring [3, 14]. Oncology teams should screen for neurotoxic chemotherapy regimens and baseline risk factors, and refer patients to physical therapy for detailed sensory, motor, and functional assessments [14]. Physical therapists play a pivotal role in administering exercise and rehabilitation interventions, collaborating extensively with oncology, nursing, and supportive care teams. Nurses are essential for ensuring adherence to protocols, educating patients, and monitoring for adverse reactions during cryotherapy [16, 18] [Moderate-certainty]. Best practices include early referral, comprehensive assessments, personalized multimodal exercise prescriptions, supervised sessions, regular outcome measurement, and interdisciplinary communication [3, 14] [High-certainty]. Acupuncture and behavioral interventions may be integrated as supplementary treatments, especially for patients with persistent symptoms or those who seek complementary therapies [20, 26][Low-certainty]. The visual abstract by Lau et al. [27], published in JAMA Neurology, illustrates the effective integration of symptoms and shared outcomes in neuropalliative care, emphasizing the significance of a multidisciplinary, patient-centered approach in optimizing outcomes for patients with CIPN (Figure 2).

**FIGURE 2:**
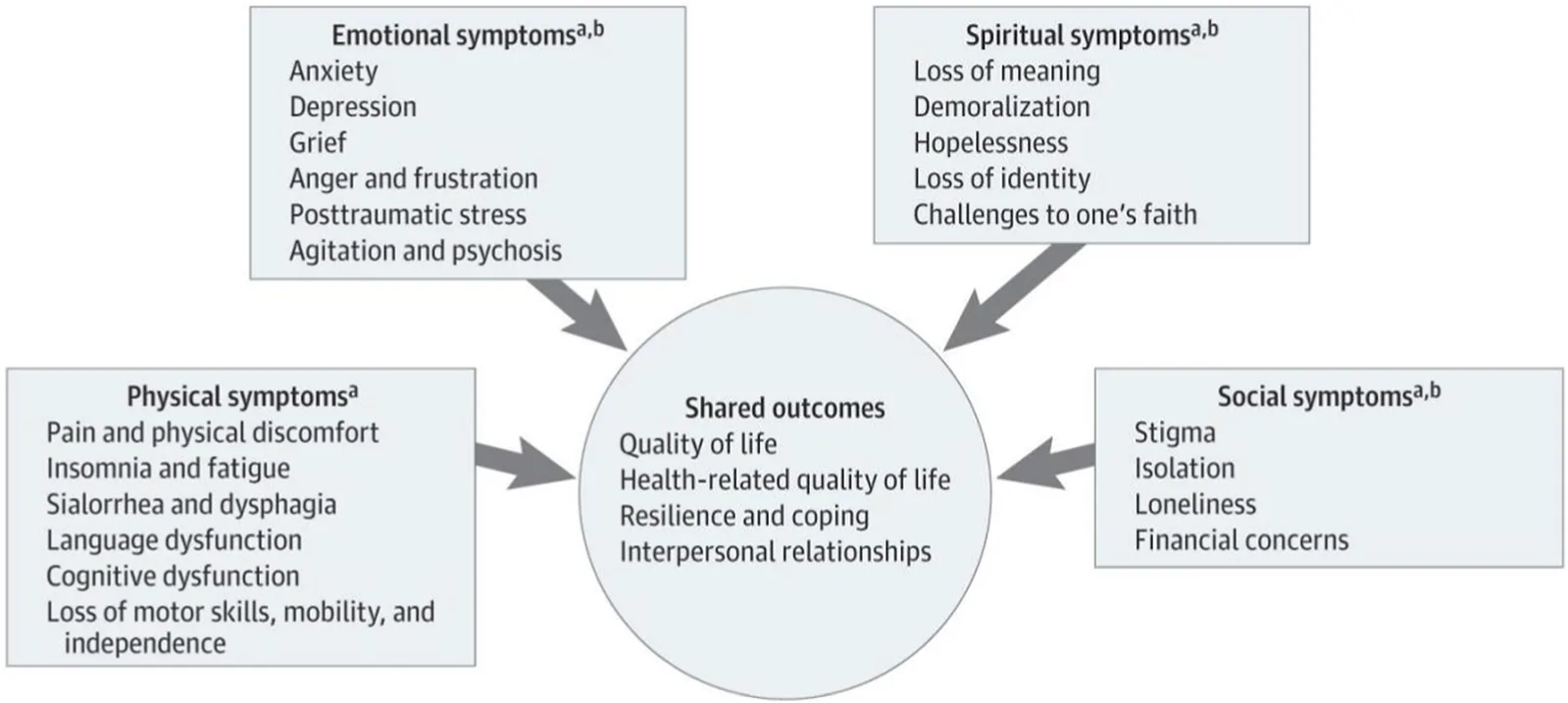
Conceptual diagram illustrating the integration of four symptom clusters (emotional, spiritual, physical, and social symptoms) and their shared outcomes (quality of life, resilience, and interpersonal relationships) in multidisciplinary neuropalliative care.

### 4.2 Facilitators for practice

Adherence to non-pharmacological interventions is a critical determinant of their effectiveness in real-world settings. A large multinational patient survey revealed that only 29% of patients employed any strategy to prevent CIPN, whereas 47% utilized exercise as a treatment strategy for established CIPN [28]. Effective strategies to enhance adherence include the development of individualized intervention plans, consistent clinical follow-up, comprehensive patient education, and the integration of behavioral support [6, 26] [Low-certainty]. Facilitators for these strategies encompass multidisciplinary collaboration, ongoing education and training, patient-centered adaptations, integration of electronic patient-reported outcome measures (PROMs), positive interpersonal dynamics, and the application of implementation science frameworks [14, 27, 29] [Moderate-certainty]. Although cost-effectiveness analyses remain limited, the existing evidence supports the benefits of multimodal exercise and cryotherapy. Multimodal exercise has been shown to reduce the incidence and severity of CIPN, enhance quality of life, and decrease healthcare utilization, all achieved with moderate resource demands and an excellent safety profile [5–7] [High-certainty]. Cryotherapy, which is low-cost and requires minimal infrastructure, has been found to significantly reduce the incidence of CIPN [16–18].

### 4.3 Barriers in practice

While adherence rates are high in structured clinical trial environments, they are compromised in routine practice due to barriers at the patient level (such as motivation, symptom burden, and comorbidities), the intervention level (including complexity, need for supervision, and duration), and the system level (such as accessibility, clinician engagement, and communication) [6, 26, 28] [Low-certainty]. Implementation barriers include the absence of standardized guidelines, gaps in provider knowledge, constraints in resources and workflow, financial challenges, and patient-level difficulties related to cognition, motivation, and cultural context [14, 29–32]System-level and patient-level barriers markedly weaken the real-world therapeutic effects of non-pharmacological CIPN interventions. Standardized clinician training and unified electronic CIPN screening workflows partially resolve implementation obstacles [Moderate-certainty]. Acupuncture’s high resource requirement limits its universal routine application for CIPN despite mild symptomatic benefits [Moderate-certainty]. Strategies to address these adherence and implementation barriers include enhancing patient education, clinician training, integration of workflows, and the utilization of electronic PROMs [30, 31, 33]. Although acupuncture may be cost-effective as an adjunct treatment for refractory symptoms, its higher resource demands and the lower certainty of evidence limit its application as a primary intervention [3, 20] [Low-certainty].

## 5 Assessment and outcome monitoring for CIPN

It is crucial to emphasize the importance of timely CIPN assessment and screening throughout the oncological treatment cycle. This includes providing a clear definition of the assessment content and selecting appropriate tools. The early identification of potential symptoms or risk factors associated with CIPN, coupled with the prompt implementation of suitable preventive and therapeutic strategies, aligns with the clinical practice guidelines proposed by the European Oncology Nursing Society [34].

### 5.1 Assessment tools for CIPN

#### 5.1.1 Chemotherapy toxicity Grading Criteria

The assessment of CIPN remains a subject of debate. Currently, the evaluation primarily depends on medical history, targeted neurological inquiry, and physical examination. Based on these components, various institutions have developed distinct rating scales, leading to a lack of standardized criteria. Commonly employed scales include the “WHO Grading Criteria” proposed by the World Health Organization [35], the “Chemotherapy Toxicity Grading Criteria” revised by the Eastern Cooperative Oncology Group (ECOG) [36], and the “National Cancer Institute-Common Toxicity Criteria” (NCI-CTC) employed by North American and Canadian oncology cooperative groups [37, 38]. Additionally, the Levi rating scale, which is specific to oxaliplatin, is utilized in some studies.

#### 5.1.2 Comprehensive neuropathy assessment tool

The Total Neuropathy Score (TNS) [39, 40] is a comprehensive assessment scale for peripheral neuropathy that systematically evaluates peripheral nerve function based on sensory symptoms, motor symptoms, autonomic symptoms, pinprick sensation, vibration sense, muscle strength, and deep tendon reflexes. Due to its comprehensiveness, it is widely used in international research for evaluating peripheral neuropathy from various causes. In recent years, international researchers have applied the TNS to the clinical diagnosis of CIPN and conducted related clinical studies. Cavaletti et al. [41–43] have demonstrated through a series of studies that the TNS is more comprehensive than various other grading standards and can be used for the clinical diagnosis and severity assessment of CIPN. However, some researchers [44] argue that this tool lacks the ability to assess the severity of neuropathic pain effectively.

#### 5.1.3 Patient-reported questionnaires

Patient-completed questionnaires are also utilized. Among these, the “FACT/GOG-Ntx” scale and the “EORTC QLQ-CIPN20” scale are frequently used in both domestic and international research [45, 46]. However, some studies [40, 47] report that both scales suffer from insufficient objectivity. Consequently, Binda et al. [48] proposed an improved questionnaire, the CIPN-R-ODS, although clinical studies on its use are still lacking.

## 6 Limitations of this narrative review

Several notable methodological limitations constrain the interpretability of the evidence synthesized in this narrative review. First, marked inter-study heterogeneity exists across non-pharmacological therapeutic protocols for chemotherapy-induced peripheral neuropathy (CIPN), with considerable discrepancies in intervention duration, training frequency, and standardized operational specifications reported among eligible clinical trials. Second, included studies deployed heterogeneous outcome measurement instruments to evaluate CIPN severity and associated symptomatology, which precludes robust quantitative pooling and head-to-head comparative analyses of intervention efficacy across cohorts. Third, trials investigating acupuncture and behavioral supportive interventions are predominantly limited by small sample sizes, substantially compromising their statistical power to draw definitive conclusions regarding clinical benefits. Finally, a subset of observational studies incorporated within this review carries substantial risks of selection and confounding bias, which downgrades the overall confidence in the evidentiary base of corresponding CIPN management strategies. To address these critical gaps in the current literature, future prospective clinical work should prioritize large-scale randomized controlled trials stratified by distinct neurotoxic chemotherapeutic agents, alongside consistent deployment of unified, validated CIPN outcome metrics; such rigorous study designs will facilitate the development of standardized, generalizable non-pharmacological intervention frameworks suitable for routine oncological supportive care.

## 7 Summary and future directions

In summary, the evidence supports multimodal exercise and physical therapy as the non-pharmacological interventions with the most robust evidence base. These interventions boast a strong safety profile and are cost-effective for both the prevention and treatment of CIPN. Cryotherapy, especially in patients with breast cancer, emerges as an exceptionally effective preventive measure. Additionally, acupuncture and behavioral interventions provide reasonable support for symptom management post-onset. The American Society of Clinical Oncology acknowledges these approaches as promising and reasonably safe but highlights the need for further research to fully understand their long-term benefits and to inform future guidelines [3]. Looking ahead, the integrated development of personalized non-pharmacological intervention plans, coupled with continuous monitoring through a multidisciplinary model that encompasses diagnosis and treatment, nursing, psychology, rehabilitation physiotherapy, and external therapies of traditional Chinese medicine, is poised to significantly enhance the outcomes for patients with CIPN.

## Data Availability

The original contributions presented in the study are included in the article/supplementary material, further inquiries can be directed to the corresponding author.
